# Supplementary material for: Antioxidant nutrition in Atlantic salmon (Salmo salar) parr and post-smolt, fed diets with high inclusion of plant ingredients and graded levels of micronutrients and selected amino acids
Source: PeerJ. 2016 Nov 8;4:e2688. doi: 10.7717/peerj.2688 (PMC5103829; doi:10.7717/peerj.2688)
Supplement: Supplemental Information 1 [file peerj-04-2688-s001.docx]

**Supplementary file:**

Hamre et al.: Antioxidant nutrition in Atlantic salmon (*Salmo salar*) parr and post-smolt, fed diets with high inclusion of plant ingredients and graded levels of micronutrients and selected amino acids.

Figure S1. Analysed concentrations of redox dependent nutrients in the diets added 0-400% NP.

Trial 1

Trial 2

Figure S2. Expression of redox related genes in liver of Atlantic salmon parr (Trial 1) and post-smolt (Trial 2) in response to dietary supplementation of micronutrients and selected amino acids at 0-400% NP. Data were first fitted to second order polynomials and the fit was then compared to a first order polynomial fit, which was chosen when p>0.05 for the second order equation. The p given in figures with first order and second order fits indicate that the slope is significantly different from 0 or that second order fits are significantly better than first order fits, respectively. n.s., not significant.
